# L'hopital Saint-Louis De Paris (1607-1911)
*This account is taken from an interesting article on the subject published in No. 14 of *Paris Médical,* from the pens of Drs. E. Gaucher and H. Gougerot.


**Published:** 1911-05-06

**Authors:** 


### L'HOPITAL SAINT-LOUIS DE PARIS (1607-1911).*

The Saint-Louis Hospital, famous alike for its connection with the past, for its architectural beauties, and for the masters who have made it the centre of French dermatology, is one of the two most ancient Paris hospitals since the disappearance of its parent establishment, the old Hôtel-Dieu, the life of which it shared. It was constructed in the reign of Henry IV., who himself laid the foundation stone of its chapel on July 13, 1607. An edict dated May 1606 charged the Governors of the Hôtel-Dieu with the task of establishing outside the city ramparts a hospital for cases of plague which hitherto had been treated along with other patients in the *Maison Dieu de Paris*. Henry wished that this lazaretto should be given the name L'Hôpital Saint-Louis, in memory of his illustrious ancestor who had died of plague in Africa, and whose tomb still exists near Carthage and is venerated by the Arabs as that of the "white marabut (or priest)."

### How It was Started.

By a proclamation he transferred "to the Hôtel-Dieu the gift and town-dues of ten sous on each minot of salt sold in the 'Generalité de Paris,' of which five sous were for fifteen years only, and five sous in perpetuity, on condition that the masters and governors should cause to be constructed a house to serve as a hospital for those sick of the contagion." A site was chosen some distance from the city walls at the foot of the hill of Belleville in the North East, between the Faubourg Saint-Martin and the Faubourg du Temple. This spot was then in the heart of the country surrounded only by the scattered homes of gardeners, and a few windmills. The building plans were drawn up by Claude Vellefaux, architect, "maçon juré aux œuvres de maçonnerie du Roy." Despite assertions to the contrary, there would seem to be no doubt that Vellefaux was the actual architect and not merely the contractor, for a document dated November 27, 1607, exists in the archives of the Hôtel-Dieu which runs as follows: "The company have delivered an order to Claude Vellefaux, juror of the King in works of masonry at Paris, for the sum of 255 livres, 3 sols, in repayment of what he has disbursed to those who have helped him to make the design and model in elevation of the maison de la Santé."

### And How It Grew.

The work made rapid progress, and by 1611 was completed. The chapel was the first to be built, while the last was the royal pavilion to-day known as the "pavillon Gabrielle," and lately disfigured in the process of enlargement. The cost of the work was 679,068 livres, 13 sols, 11 deniers, or more than £80,000 of present-day currency. At that time the entrance was near the chapel, through the pavilion, which is still surmounted by the bust of the good King its founder, but this arched passage has since been walled up. On either side of this entrance there stood, to the North a dispensary, and to the South a kitchen. The latter retains its position to this day.

The main block of buildings formed a large square, and was devoted to charity patients. Around this central structure ran a wide pathway which in turn was surrounded by a wall. Sentinels were placed on the last-named to prevent communication between the inmates and the outside world. At each of the four corners of the outside walls buildings were erected. Those on the eastern side accommodated paying patients, while those on the western side near the old entrance housed the working staff. These last two pavilions were joined up to the main central block by two covered galleries, of which one still exists near the pharmacist's quarters. A third covered gallery, starting from the middle of the west front, made communication possible between the sick wards and the entrance pavilion, and thence between them and the kitchen and dispensary. The central buildings could be approached from the surrounding pathways by stone stairs, which no longer exist. The lofty and beautiful rooms of the first floor alone were originally intended to house the sick, and it was not until the nineteenth century that the low rooms of the ground floor were put to this use. Access to the central court was obtained through a narrow passage. Save for certain badly designed additions and ill-executed repairs this court has come down to us intact. It alone was open for the patients to walk about in, and everything else was closed, grilled, bolted, and barred, so that the place was a veritable lazaretto. To such an extent was Saint-Louis isolated that in 1639 an edict provided that steps should be taken to defend it: "This said day it is ordained that there shall be purchased halberds and other arms to be taken to Saint-Louys for it to guard itself against thieves."

### Its Cases.

For more than half of the seventeenth century Saint-Louis received cases or suspected cases of plague from the Hôtel-Dieu, but was open only in the years of epidemics. The administrative work was carried on by the doctors and nuns attached to the mother-house. When, as the result of severe police regulations and edicts of Parliament, the plague was seen no more, the new institution opened its gates to the convalescents of the Hôtel-Dieu, and from 1670 to 1690 scorbutic patients were sent to it from Bicêtre and la Salpêtrière also. During the times of misery and famine which marked the close of the reign of Louis XIV. it was made use of as a lodging-house for beggars and as a storehouse for corn. Like the Hôtel-Dieu, it reflected accurately the state of society in Paris. Each period of activity marked a time of hardship in the city. For twenty years from 1709 it closed its doors, and it was seriously proposed to devote it to other uses. The energetic protests of the Governors of the parent institution saved it, however, and it was kept for emergencies as a hospital, a prudent course which subsequent events amply justified.

### Further History.

In 1729 it was again opened for scorbutic patients, and in 1737 it housed those inmates whom a fire at the Hôtel-Dieu rendered homeless. In 1749 the wards of the ground floor were ordered to be set apart for the reception of able-bodied beggars. From 1754 to 1772 the institution was closed, but in the latter year a second fire at the Hôtel-Dieu caused its doors to be opened once more, and since then it has carried on its work without interruption. Being far from the centre of the city, its physicians were not called upon to make it a daily visit. Consequently only incurable and chronic ailments were treated within its walls. Little by little it became the recognised place for the reception and treatment of skin diseases, until in 1801 a decree of the Administrative Council of the Hospitals lent official sanction to its claims as a special hospital; for the Hôpital du Nord (the name given it after the Revolution) was declared to exist "to receive chronic maladies, whether contagious, such as

* This account is taken from an interesting article on the subject published in No. 14 of *Paris Médical*, from the pens of Drs. E. Gaucher and H. Gougerot.

scabies, ringworm of the scalp, or rebellious and cachetic, such as skin diseases, scurvy, ulcers, scrofula."

Its Rise as a Skin Hospital.

It was the genius of Alibert, who had been appointed chief physician to the hospital in 1803, that quickly made it the great skin hospital of the world. Among the long list of physicians who have worked within its walls, the names of Alibert and Bazin stand out pre-eminently. The former was the first to introduce some semblance of order out of the chaos which hitherto had reigned in the world of dermatology. He showed how to classify ulcers into syphilitic, scrofulous, herpetic, etc., and was the first to treat skin diseases from the objective as contrasted with the empiric standpoint which hitherto had obtained. The first clinical professor to be appointed, he gave his lectures in the open air, *sub Jove*, after the fashion of the ancient Greek philosophers. Mounted on a platform, having on one side his tree of skin diseases which he had imitated from the botanical classification of Jussieu, and on the other the patient upon whom he was demonstrating, he held the attention of his audience by sheer personal charm. Unfortunately, he left no worthy disciple behind him, for in his general ideas he was head and shoulders above his contemporaries, and few, if any, of them understood him. Biett, his favourite pupil, after being nominated assistant to the hospital, became his rival, his adversary, and almost his enemy. Lugol, Cazenave, Gibert, and Devergie followed in quick succession, but none of them was prolific in original ideas, and it was not until Bazin entered the hospital in 1850 that a successor worthy of Alibert was found. It may be said, and with truth, that if the works of this great observer had been read and understood we should have been spared many modern "discoveries." His contemporary, Hardy, was to a certain extent his rival also. Although on the staff of a special hospital he continued to practise as a general physician, and was fond of saying that the good specialist must be the product of evolution, or, in other words, that he must be a specialised physician. Bazin and Hardy have been followed in turn by Hillairet, Laillier, Guibout, Vidal, Fournier, Hallopeau, Quinquad, Tennesson, Du Castel, and Danlos, all of whom are dead or retired by reason of the age-limit. Fournier was the founder of the official teaching in dermatology and syphiligraphy in France and the first incumbent of the chair of Clinical Medicine at the Saint-Louis Hospital.

